# The burden of stroke in Brazil in 2016: an analysis of the Global Burden of Disease study findings

**DOI:** 10.1186/s13104-018-3842-3

**Published:** 2018-10-16

**Authors:** Nathalia Matos de Santana, Francisco Winter dos Santos Figueiredo, Diego Monteiro de Melo Lucena, Fernando Mayo Soares, Fernando Adami, Luciana de Carvalho Pádua Cardoso, João Antonio Correa

**Affiliations:** 1 Faculdade de Medicina do ABC, Santo André, SP Brazil; 2 Laboratório de Epidemiologia e Análise de Dados, Faculdade de Medicina do ABC, Santo André, SP Brazil; 3 Disciplina de Angiologia e Cirurgia Vascular, Faculdade de Medicina do ABC, Santo André, SP Brazil

**Keywords:** Stroke, Epidemiology, Brazil

## Abstract

**Objective:**

To analyze the epidemiological stroke data of Brazil according to the Global Burden of Disease (GBD) study in 2016 and secondary data from the GBD database.

**Results:**

The highest percentage of deaths due to stroke in general occurred in individuals aged 70 years or over (60.2%; 95% confidence interval [CI] 59.9–60.5%) followed by that in men (52.9%; 95% CI 52.6–53.2%). Ischemic stroke was the most common type, accounting for 61.8% (95% CI 61.5–62.1%) of deaths due to stroke in 2016. Most of the epidemiological indicators (incidence, prevalence, mortality-to-incidence ratio, mortality, disability-adjusted life years, years lost due to disability, and years of life lost) of stroke in general or either type of stroke were higher in men and those aged 70 years or over. Stroke data in Brazil are a major concern and represent a real health challenge for the coming decades. Men and individuals aged 70 years or older appear to represent the groups with the highest epidemiological parameters and risk for the various stroke outcomes. However, this does not mean the female data are irrelevant, which, although representing a lower risk than the male data, also raise the need for policies aimed at prevention and improvement in the treatment of stroke and its sequelae.

## Introduction

Developed countries have experienced a drop in the indicators of mortality due to stroke in recent decades, in contrast to low- and middle-income countries that have shown an increasing trend of morbidity and mortality from this disease [1–3]. Brazil, however, has experienced a downward trend in stroke mortality, albeit at a slower rate than that of developed countries [4–6].

Emerging countries such as Brazil [7] have shown developments and improvements in access to education and technology as well as improvements in primary health care, with an emphasis on prevention [2]. The application of these practices has resulted in an increase in life expectancy, changes in lifestyle, and consequently changes in the way people live and take care of their health [8, 9].

With these changes, the control, prevention, and treatment of risk factors have been prioritized to decrease mortality due to cardiovascular and cerebrovascular diseases such as stroke [10–12]. In 2010, stroke was considered the leading cause of death [13] and the third cause of disability-adjusted life years (DALYs) [14] worldwide.

However, owing to the rapid changes in the behavior of diseases, it is essential to update epidemiological studies for the adequate formulation of public policies, especially regarding epidemiological parameters related to disease burden. In this context, the objective of the present study was to describe the epidemiological aspects of stroke in Brazil in 2016.

## Main text

### Methods

#### Study design

This was a descriptive study with data from the Global Burden of Disease (GBD) study conducted in Brazil in 2016.

#### Data source

To understand the epidemiological aspects of diseases in different parts of the world, including Brazil, GBD researchers developed statistical tests to estimate the global burden of 291 diseases in all countries divided into 21 groups [15].

The GBD database is maintained by the Institute for Health Metrics and Evaluation at the University of Washington in Seattle, Washington, USA (http://www.healthdata.org).

The data sources used to construct the GBD database include representative national surveys, cancer registries, vital records systems, and health care use data and encompass 291 diseases in all countries of the globe separated into 21 groups [15].

Information about GBD data can be found in the GBD Data Input Sources Tool in the GHDx (http://ghdx.healthdata.org/gbd-2016). The tool allows users to explore the input sources to GBD based on various criteria, and to export and explore the results. The GBD database is maintained by the Institute for Health Metrics and Evaluation at the University of Washington in Seattle, Washington, USA (http://www.healthdata.org).

For Brazil, the main source of mortality data used in the GBD study was the database of the Mortality Information System of the Ministry of Health, adjusted for other national and international sources.

Therefore, this database provides a comprehensive annual assessment of consistent estimates of health loss due to mortality and morbidity of diseases, injuries, and risk factors through approaches, statistical modeling, and metrics, such as incidence, prevalence, mortality, years of life lost (YLL) due to premature mortality, mortality-to-incidence ratio (MIR), years lived with disability (YLD), disability-adjusted life years (DALYs), and health-adjusted life expectancy.

The GBD study uses rigorous methods to nullify biases in the sources and models used to generate estimates based on the complexity of disease epidemiology. In addition, advanced statistical methodologies are used to estimate regional results, thus reducing sampling bias. The results are updated annually for the entire time series and these results supersede earlier versions of the GBD study.

#### Variables studied

The variables studied related to stroke were Incidence, Prevalence, Mortality, mortality-to-incidence ratio, YLL, YLD, and DALYs overall and according to sex, age range (15–49, 50–69, and ≥ 70 years), and type of stroke (ischemic or hemorrhagic) occurring in Brazil in 2016. These data are expressed as number per 100,000 inhabitants. The indicators studied are presented in Table 1.

**Table 1:** Epidemiological indicators, calculations, and interpretation of the results of the indicators

Indicator	Calculation	Interpretation
Mortality	No. of deaths/population × 100,000	Proportion of people who die of a disease
Incidence	No. of new cases detected during a given time period × 100,000/No. of persons at risk of developing the disease during the same period	Number of new cases of a given disease over a defined period in a population at risk of developing the disease
Prevalence	Number of known cases of the disease in a given period × 100,000/population during the same period	Proportion of people in a given population that present a specific disease at a particular point in time
MIR	Mortality rate/incidence rate	Indicator used to estimate survival of any disease in 5 years
DALYs	YLL + YLD	Years of healthy life lost
YLD	Number of incident cases x weight of disability x average duration until remission or death	The number of years that an individual lives with a functional impairment caused by an illness
YLL	Number of deaths x standard life expectancy at the age of death	The number of years a person could have lived if the illness that killed him had not occurred

*DALYs* disability-adjusted life years, *MIR* mortality-to-incidence ratio, *YLD* years lived with disability, *YLL* years of life lost

#### Data analysis

Data are presented as absolute frequency and relative frequency. To estimate the mortality-to-incidence ratio (MIR), the Mortality and Incidence rates for stroke were standardized for age and expressed per 100,000 people. The statistical program used to analyze the data was Stata^®^ version 11.0 (StataCorp LLC, College Station, TX, USA).

### Results

In Brazil in 2016, there were 107,258 deaths due to stroke, with the highest numbers observed in men (56,782 deaths, 52.9%, 95% confidence interval [CI] 52.6–53.2%) and in the group of individuals over 70 years of age (64,582 deaths, 60.2%, 95% CI 59.9–60.5%). The ischemic type of stroke accounted for the most deaths (66,261 deaths, 61.8%. 95% CI 61.5–62.1%). Regarding the burden of stroke per 100,000 Brazilian inhabitants in 2016, there was an incidence of 138.91, mortality of 63.15, 1437.74 DALYs, 14.77 YLD, and 1337.74 YLL (Table 2).

**Table 2:** Deaths and epidemiologic indicators due to stroke in Brazil in 2016

Characteristic	n	% (95% CI)
Total	107,258	100,00
Sex		
Male	56,782	52.9 (52.6; 53.2)
Female	50,476	47.1 (46.8; 47.4)
Age group, years		
15–49	9332	8.7 (8.5; 8.9)
50–69	33,344	31.1 (30.8; 31.4)
≥ 70	64,582	60.2 (59.9; 60.5)
Type of stroke		
Ischemic	66,261	61.8 (61.5; 62.1)
Hemorrhagic	40,997	38.2 (37.9; 38.5)
Epidemiologic indicators	Value (per 100,000 inhabitants)	
Incidence	138.91	
Prevalence	1008.02	
Mortality	63.15	
MIR	0.06	
DALYs	1437.74	
YLD	14.77	
YLL	1331.74	

*CI* confidence interval, *DALYs* disability-adjusted life years, *MIR* mortality-to-incidence ratio, *YLD* years lived with disability, *YLL* years of life lost

Regarding the burden of stroke according to sex and type of stroke, higher epidemiological indicators were observed among men compared with women, except for the prevalence of hemorrhagic stroke and the number of YLD, which were higher in women (Table 3).

**Table 3:** Stroke epidemiology according to stroke type and sex in Brazil in 2016

Indicators	Sex		Age group		
	Male	Female	15–49 years	50–69 years	≥ 70 years
Overall					
Incidence	160.13	122.9	32.78	298.58	971.78
Prevalence	1153.94	897.44	264.9	2267.69	6816.73
Mortality	80.68	51.15	8.2	88.7	599.9
MIR	0.50	0.41	0.25	0.039	0.09
DALYs	1437.74	961.87	403.58	2587.61	7160.72
YLD	106	99	23.92	196.36	783.89
YLL	1331.74	862.87	379.66	2391.25	6376.83
Ischemic					
Incidence	120.6	85.16	403.58	2587.61	7160.72
Prevalence	1018.7	731.22	207.38	1857.22	6085.24
Mortality	54.17	32.48	1.27	41.59	456.83
MIR	0.44	0.04	0.0031	0.022	0.075
DALYs	814.66	490.28	72.75	1215.72	5393.9
YLD	91.22	78.38	16.73	157.01	681.21
YLL	723.44	411.9	56.02	1058.71	4712.69
Hemorrhagic					
Incidence	39.53	37.74	13.68	87.64	218.08
Prevalence	170.99	201.64	83.57	453.29	901.91
Mortality	26.51	18.68	6.93	47.1	143.1
MIR	0.67	0.09	0.50	0.1	0.16
DALYs	623.08	471.59	330.83	1371.89	1766.82
YLD	14.77	20.62	7.19	38.34	102.68
YLL	608.31	450.97	323.64	1333.55	1664.14

*DALYs* disability-adjusted life years, *MIR* mortality-to-incidence ratio, *YLD* years lived with disability, *YLL* years of life lost

Regarding the burden of stroke according to age group and type of stroke, the majority of epidemiological indicators were higher in the age group of 70 years or older. However, the MIR was higher among individuals aged 15–49 years (MIR = 0.25) than that observed in those aged 50–69 years (MIR = 0.04) or 70 years or older (MIR = 0.09) for the general stroke category as well as the hemorrhagic subtype (15–49 years, MIR = 0.50; 50–69 years, MIR = 0.10; ≥ 70 years, MIR = 0.16) (Table 3).

### Discussion

When describing the burden of Stroke in Brazil in 2016, it was observed that:

i). Most of the epidemiological indicators of stroke burden are higher in men than in women.
ii). Women have a higher prevalence of hemorrhagic stroke and more years of disability (YLD) than men.
iii). Both stroke in general and hemorrhagic subtype present higher mortality indicators in individuals aged 15–49 years.

The Stroke Association reported in 2018 [16] that men are more likely to experience a stroke than women because men are more prone to alcoholism and smoking, which are important risk factors for cardiovascular and metabolic diseases.

In the period between 2005 and 2015, the trend of stroke mortality was constant, with a higher number of deaths from the disease in 2007 and 2008, affecting more men, individuals over 65 years of age and white individuals [17].

Studies conducted by the National Health Survey (2013) revealed that stroke mostly affects older people, those without a formal education, and residents of urban centers. In addition, more men than women are affected by stroke leaving functional disabilities in this public [18]. An addition, men are still considered to comprise the majority of people affected by stroke [19].

However, the present study observed that women had a higher prevalence of hemorrhagic stroke and more YLD than men. The high chance of hemorrhagic stroke in women has already been documented in some studies, including in Latin American countries. Diaz et al. demonstrated a high incidence of hemorrhagic stroke in females associated mainly with alcohol intake, smoking, hypertension and dyslipidemia [20, 21].

At the same time, women have some unique risk factors, such as hormonal changes caused by hormonal contraception, pregnancy, childbirth, and hormone replacement therapy [22]. In this regard, many studies show that the prevalence is higher in women, as found in the present study.

Women have a higher YLD compared to men in hemorrhagic stroke, which could also give an indication of this important female increase in the epidemiology of this type of stroke The YLD reflects one of the most alarming data on stroke. Individuals 70 and older have worse indices in all groups and types of stroke studied, which shows the urgency of the need for care and follow-up of this population after stroke. In addition, there are differences between sex and type of stroke [20, 21].

In Brazil, aging is the strongest non-modifiable risk factor for ischemic stroke, and elderly stroke patients have higher mortality and morbidity and worse functional recovery than younger patients [23]. Although high cerebrovascular accident rates occur in individuals older than 70 years, in 2016, both stroke in general and hemorrhagic subtype present higher mortality indicators in individuals aged 15–49 years [24].

Stroke in individuals over 70 years of age is a disabling disease, related to a longer period of living with sequelae and causing the burden of disease to be higher in this specific age group [25], as found in the present study.

Data on DALYs reflect a greater loss of economic productivity in both sexes and stroke types, but with more significant numbers in men aged 50–69 years in which they are still economically active. At the same time, the YLL numbers reinforce the thesis that men still lose many more years of life due to ischemic stroke, but this difference decreases in relation to females when we discuss hemorrhagic stroke. This could be explained by the considered increase of cases of this modality of disease in the women in the last years [20, 21].

In contrast to Brazilian statistics, in other countries, stroke is more common in younger individuals and in individuals in economically active age groups [4]. The Stroke Association [16] reported that in countries such as the United States and the United Kingdom, the rate of stroke for the first time in people aged 45 years or older is expected to increase over the next 20 years.

Cardiovascular disease mortality in Brazil is one of the highest among the Latin American countries, and although this mortality rate has declined in recent years (between 1980 and 2002), the decrease has not been consistent different regions, and the Northeast still shows higher rates [17, 18].

In addition, Brazil is a country with a vast territorial extension and cultural plurality that undergoes constant socioeconomic and political changes. In this sense, other epidemiological studies that consider socioeconomic, demographic, and cultural aspects in a longitudinal design should be encouraged.

In Brazil, in 2016, the burden of stroke affected more men and individuals over the age of 70 in general, but the prevalence of hemorrhagic stroke was more striking in women. It is also women who experience the most disabilities, although men die more frequently. In addition, stroke has a high lethality in individuals aged 15–49 years.

The differences in morbidity and mortality from stroke are not only observed in Brazil, but in most Latin American and developing countries. Thus, each country should take into account the prevention of the classic risk factors for cardiovascular diseases, respecting the epidemiological, cultural and socioeconomic realities of each region. This would be possible through greater regional freedom to create and implement prevention programs. In Brazil, for example, this would be possible through the Basic Health Units. Coverage has been increasing in recent years and it is possible to become an instrument for the promotion and prevention of diseases with greater epidemiological importance, as is already done with breast cancer and of cervix of uterus [26, 27].

## Limitations

The limitations of this study are its descriptive nature and the fact that it is only a snapshot in time and not a longitudinal analysis.

## Abbreviations

GBD: Global Burden of Disease
DALYs: disability-adjusted life years
MIR: mortality-to-incidence ratio
YLD: years lived with disability
YLL: years of life lost
